# Gelatin/Hyaluronic Acid Photocrosslinked Double Network Hydrogel with Nano-Hydroxyapatite Composite for Potential Application in Bone Repair

**DOI:** 10.3390/gels9090742

**Published:** 2023-09-13

**Authors:** Jianuo Zheng, Yunping Wang, Yuwen Wang, Ruiping Duan, Lingrong Liu

**Affiliations:** Tianjin Key Laboratory of Biomedical Materials, Institute of Biomedical Engineering, Chinese Academy of Medical Sciences and Peking Union Medical College, Tianjin 300192, China; zhengjianuo0118@163.com (J.Z.); pumcwyp@163.com (Y.W.); yuwenw2023@163.com (Y.W.); rpduan@sina.com (R.D.)

**Keywords:** gelatin, hyaluronic acid, nano-hydroxyapatite, double network hydrogel, injectable hydrogel

## Abstract

The application of hydrogels in bone repair is limited due to their low mechanical strength. Simulating bone extracellular matrix, methylacrylylated gelatin (GelMA)/methylacrylylated hyaluronic acid (HAMA)/nano-hydroxyapatite(nHap) composite hydrogels were prepared by combining the double network strategy and composite of nHap in this study. The precursor solutions of the composite hydrogels were injectable due to their shear thinning property. The compressive elastic modulus of the composite hydrogel was significantly enhanced, the fracture strength of the composite hydrogel nearly reached 1 MPa, and the composite hydrogel retained its high water content at above 88%. The composite hydrogels possess good compatibility with BMSC_S_ and have the potential to be used as injectable hydrogels for bone defect treatment.

## 1. Introduction

Bone defects caused by severe trauma, bone tumors, or congenital disorders have become diseases that seriously threaten human health [1]. Bone is a dynamic, innervated, and vascularized tissue with the ability to regenerate after injury. However, for larger bone defects, bone implants are needed to facilitate complete bone healing [2]. Current methods for repairing bone defects include autologous bone grafting (considered the gold standard), allografts, and xenografts. Both autografts and allografts are limited by donor tissue availability and donor site morbidity. Additionally, both allografts and xenografts present issues related to cost and disease transmission [3,4]. Therefore, there is a need to develop artificial bone repair materials with good biocompatibility and osteoinductivity for the treatment of bone defects [5,6].

The extracellular matrix is a complex network of various biomacromolecules that plays an important role in maintaining tissue structure and regulating cellular function and activity. Hydrogels are three-dimensional crosslinked macromolecule networks that can absorb and retain a large amount of water. Due to their physical and chemical properties being similar to the extracellular matrix, many types of hydrogels have been applied in tissue engineering and tissue regeneration [7,8]. Among the hydrogels used for tissue repair, injectable hydrogels have distinct advantages and great potential in clinical treatment. Because of their sol–gel property, injectable hydrogels can be implanted into the body through minimally invasive surgery, avoiding traditional major surgery, and also possess good spatial adaptability for repairing irregularly shaped wounds [9,10].

Bone is a hard tissue with a certain mechanical strength as a mechanical support and protection of organisms. However, the application of hydrogels for bone repair is limited due to their low mechanical strength. Many design strategies and preparation techniques of hydrogels, such as the multi-crosslink strategy and nanoparticle composite design, have been applied to improve the mechanical properties of hydrogels [9]. The hydrogels for medical purposes are usually formed by a single crosslinked macromolecule network. Although the mechanical strength of hydrogels can be enhanced by increasing the crosslink degree, the brittleness of hydrogels is increased at the same time, which leads to the insufficient mechanical properties of single network (SN) hydrogels. The double network (DN) hydrogels consist of two interpenetrating crosslinked macromolecule networks with different chemical structures and mechanical properties: one is a rigid network that dominates the elastic modulus of hydrogel; the other is a soft and stretchable molecular chain network. The DN hydrogel strategy has been used to improve the mechanical strength of hydrogel and to adjust the components and properties of hydrogel [10,11]. Recently, we developed a gelatin/hyaluronic acid DN hydrogel; this DN hydrogel possess enhanced and adjustable mechanical strength with higher cell viability, good cell compatibility, and promoted rabbit bone mesenchymal stem cells (BMSCs) cartilage differentiation [12,13].

Bone extracellular matrix is mainly composed of inorganic and organic components, consisting of 50–70% inorganic components (mainly hydroxyapatite), 20–40% organic components (mainly type I collagen), 5–10% water, and 3% lipids. Hydroxyapatite is one of the components of bone tissue with good biocompatibility, osteoinduction, and osteointegration, providing the mechanical strength of bone tissue. Nano-hydroxyapatite (nHap) deposits and mineralizes on collagen fibers to form bone [14]. By incorporation of different nanoparticles, the physicochemical properties of hydrogels such as viscosity, stiffness, viscoelasticity, and surface properties can be tuned [15]. Especially, the mechanical strength of nanocomposite hydrogels can be improved and adjusted [16]. Due to the nature of the nanoparticle, the biological properties of nanocomposite hydrogels are also changed. As the main inorganic component of bone, the chemical and structural properties of nHap are similar to the inorganic phase of bone, therefore nHap composite materials exhibit good integration with the surrounding bone, bioactivity, and osteoconductive properties, and promote differentiation of stem cells leading to osteogenesis [16,17].

For simulation of the bone extracellular matrix, gelatin, hyaluronic acid, and nHap were used for injectable composite DN hydrogel for bone repair in this study [15,18]. Gelatin is the product of the partial hydrolysis of collagen, which is the main component of extracellular matrix. It possesses an amino acid composition similar to collagen, such as arginyl-glycyl-aspartic acid (RGD)—a tripeptide with bioactivities that promote cell adhesion, proliferation, and differentiation—and certain matrix metalloproteinase sequences that can be degraded by collagenase [19,20]. So, gelatin is a hydrophilic macromolecule with good biocompatibility and biodegradation. Hyaluronic acid is a linear polysaccharide macromolecule and one of the important GAGs in bone tissue, especially in cartilage [21]. Moreover, hyaluronic acid can be degraded by hyaluronidase enzymes [21,22]. The amino group of the gelatin molecular chain, as well as the hydroxyl group and carboxyl group of the hyaluronic acid molecule, are both targets for chemical modification to improve their physicochemical properties. Through methylacrylylation modification, a methylacrylylated gelatin (GelMA) and methylacrylylated hyaluronic acid (HAMA) solution can be photocrosslinked by UV or visible light to form a hydrogel [23]. This kind of sol–gel transition is suitable for application in injectable hydrogels, which can be implanted in vivo by minimally invasive surgery [24].

In this study, GelMA/HAMA/nHap composite hydrogels were prepared and characterized, the rheological property of the precursor solution for hydrogels was tested for injectability, and the mechanical properties of the hydrogels and in vitro cytocompatibility of the hydrogels were evaluated for their application in bone repair.

## 2. Results and Discussion

### 2.1. Preparation of GelMA/HAMA/nHap Composite Hydrogels

**Figure 1:**
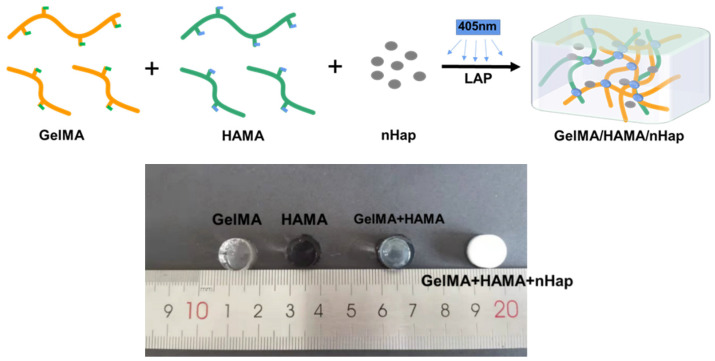
The preparation scheme of GelMA/HAMA/nHap composite hydrogel.

The preparation scheme of the nHap composite photocrosslinked gelatin/hyaluronic DN hydrogel is shown in Figure 1. The GelMA/HAMA/nHap composite hydrogels were prepared by uniformly distributing nHap into the GelMA/HAMA double network followed by photocrosslinking. Gelatin and hyaluronic acid are widely applied for biomedical materials for their good biocompatibility and biodegradability [19,22]. In recent years, many methylacrylylated biomacromolecules have been synthesized and applied for the preparation of photocrosslinked hydrogel. GelMA and HAMA are methylacrylylated gelatin and hyaluronic acid, which can be crosslinked to form hydrogel quickly by UV and visible light under the action of a photoinitiator and are suitable for preparing an injectable hydrogel [20]. Both the GelMA and HAMA photocrosslinked hydrogels have promising application prospects as biomedical materials [23,24]. However, being SN hydrogels, GelMA and HAMA photocrosslinked hydrogels both have insufficient mechanical characteristics; so, the strategy of DN hydrogel is used to improve such characteristics. nHap particles have the excellent property of promoting osteogenesis, and the composite of nHap can also enhance the mechanical strength of the hydrogel [25,26]. The blue initiator LAP and 405 nm visible light were used for photocrosslinking of the composite hydrogel in our study. Compared with UV photocrosslinking, visible light photocrosslinking is mild and may be beneficial to the cytocompatibility of the photocrosslinked hydrogel.

**Figure 2:**
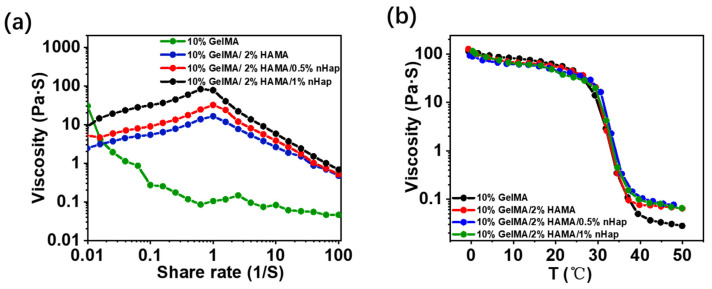
Rheological properties of hydrogels: (**a**) viscosity scan; (**b**) temperature scan.

Since the GelMA/HAMA/nHap composite hydrogels are intended for use in bone repair by injection in vivo, the rheological properties of the hydrogels should be tested to determine their injectability. The shear thinning property of the fluid, which is defined as the viscosity of the fluid decreasing with increasing shear rate, indicates the injectability of the fluid [27,28]. Figure 2a shows the change in viscosity of the precursor solution for hydrogels with the increasing of shear rate at 25 °C. For the precursor solution of 10%GelMA hydrogel, the viscosity decreased as shear rate increased at a low shear rate, and there was no obvious shear thinning property at a high shear rate. For the precursor solution of 10%GelMA/2%HAMA, 10%GelMA/2%HAMA/1%nHap, and 10%GelMA/2%HAMA/0.5%nHap hydrogels, the viscosity increased as shear rate increased when the shear rate was lower than 1/S, and the viscosity decreased as shear rate increased when the shear rate was higher than 1/S. The results showed that for the precursor solutions of 10%GelMA/2%HAMA, 10%GelMA/2%HAMA/1%nHap, and 10%GelMA/2%HAMA/0.5%nHap hydrogels, there were no obvious shear thinning characteristics at a low shear rate but there was obvious shear thinning at a high shear rate over 1/S. As shown in Figure 2b, all precursor solutions of hydrogels changed from high viscosity to low viscosity with the increasing of temperature, and the transition temperature range was from 25 °C to 34 °C, which is attributed to the temperature sensitivity of gelatin [29,30]. The precursor solutions of GelMA/HAMA hydrogel and nHap composite GelMA/HAMA hydrogels all showed a shear thinning property due to hyaluronic acid, the solution of which presents shear thinning and is often used as an injectable reagent in the field of biomedicine [31]. The shear thinning property and temperature sensitivity of the precursor solution of hydrogels are desirable to improve the uniform distribution of nHap in the mixture by changing the parameters of the preparation process such as stirring speed and temperature. The shear thinning property of the precursor solution of hydrogels indicates that it has good injectability; so, the precursor solutions of the composite hydrogels are injectable.

### 2.2. Characterization of GelMA/HAMA/nHap Composite Hydrogels

**Figure 3:**
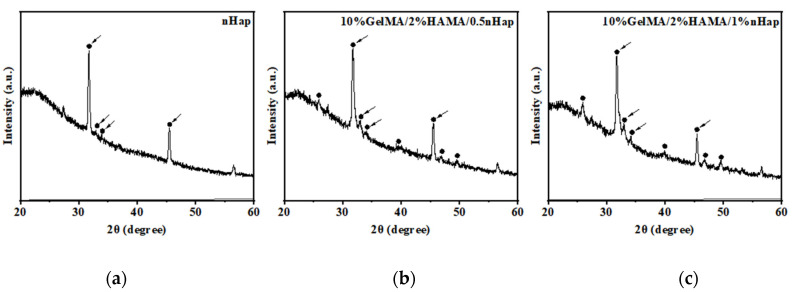
XRD spectra of (**a**) nHap, (**b**) 10%GelMA/2%HAMA/0.5%nHap hydrogel, and (**c**) 10%GelMA/2%HAMA/1%nHap hydrogel; arrows point to hydroxyapatite characteristic peaks.

The prepared GelMA/HAMA/nHap composite hydrogels were characterized by X-ray photoelectron spectroscopy (XPS) and X-ray diffraction spectrum (XRD) analytical methods. In Table 1, the results of surface chemical elements analysis from XPS of the hydrogels show that the Ca and P element ratio increased with the increase in nHap ratio in GelMA/HAMA/nHap composite hydrogels from 0.5% to 1%. As shown in Figure 3, the XRD spectrum of nHap presents the typical hydroxyapatite crystal characteristic peak [32], while the XRD spectra of 10%GelMA/2%HAMA/0.5%nHap and 10%GelMA/2%HAMA/1%nHap hydrogels contained the peak characteristic for hydroxyapatite crystal compared with the one of nHap. The results above support the combination of nHap and GelMA/HAMA DN hydrogels.

**Figure 4:**
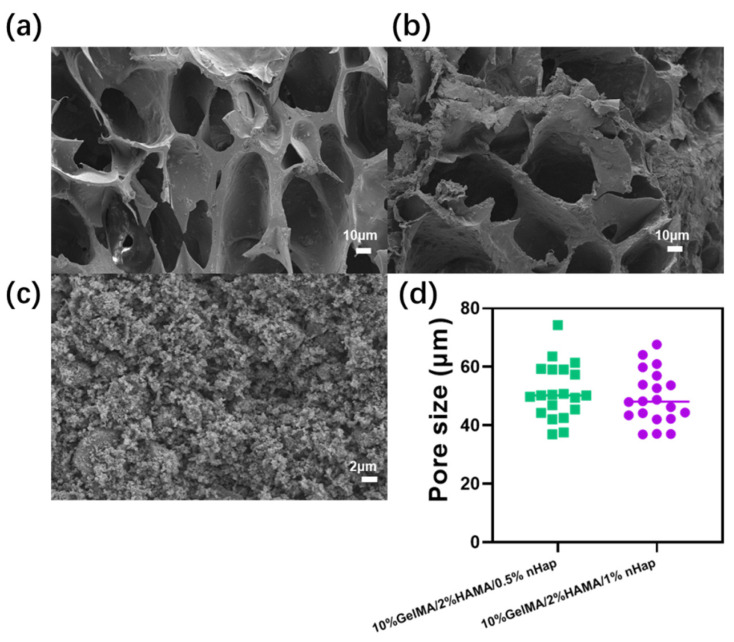
SEM image of (**a**) 10%GelMA/2%HAMA/0.5%nHap hydrogel, scale bar = 10 μm; (**b**) 10%GelMA/2%HAMA/1%nHap hydrogel, scale bar = 10 μm; and (**c**) nHap, scale bar = 2 μm. (**d**) The distribution of hydrogel pore size.

After freeze-drying of the samples, the microstructure of the hydrogels was observed by scanning electron microscope (SEM). According to Figure 4, the 10%GelMA/2%HAMA/0.5%nHap and 10%GelMA/2%HAMA/1%nHap hydrogels were both connected porous structures after they were freeze-dried, and the pore sizes of 10%GelMA/2%HAMA/0.5%nHap and 10%GelMA/2%HAMA/1%nHap hydrogels were 51.14 ± 9.21 μm and 49.41 ± 8.85 μm, respectively. At the same time, nHap was wrapped on and adhered to the pore wall, and no nHap particle aggregation was observed, indicating that nHap was uniformly dispersed in the composite hydrogel.

### 2.3. Compressive Strength of GelMA/HAMA/nHap Composite Hydrogels

**Figure 5:**
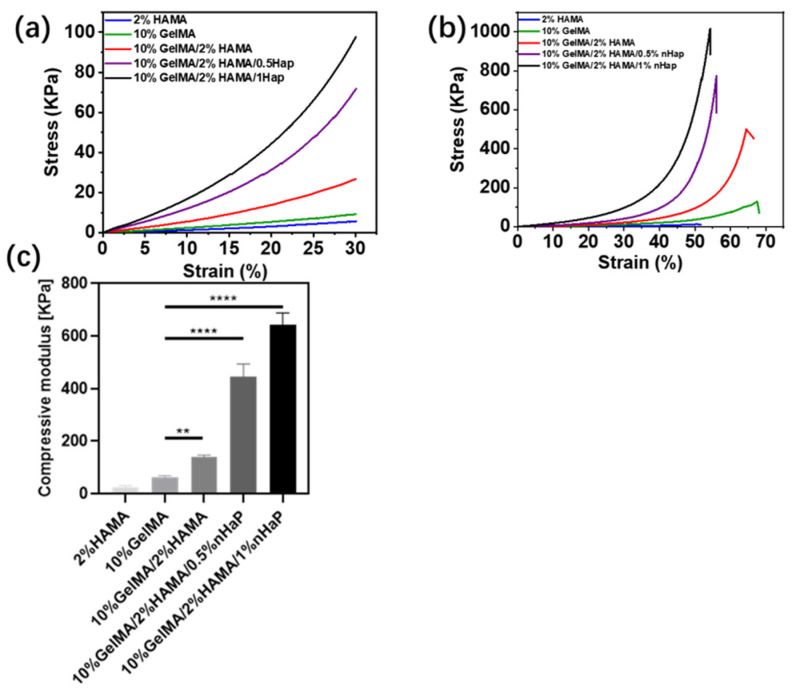
(**a**) The stress–strain curves of the hydrogels in the 0–30% strain region; (**b**) the stress–strain curves of the hydrogels until the sample broke; and (**c**) compressive modulus of the hydrogels. Data are expressed as mean ± SD (*n* = 3; ** *p* < 0.05, **** *p* < 0.0001).

Hydrogels for bone repair require sufficient mechanical properties, such as compressive strength [9,33]. As shown in Figure 5a,c, the compressive elastic moduli of the 2%HAMA and 10%GelMA SN hydrogels were 24.36 ± 4.79 KPa and 62.74 ± 4.55 Kpa, respectively; the compressive elastic modulus of the 10%GelMA/2%HAMA DN hydrogel was 141.06 ± 5.01 Kpa; and those of the 10%GelMA/2%HAMA/0.5%nHap and 10%GelMA/2%HAMA/1%nHap composite hydrogels were 446.34 ± 38.43 KPa and 644.25 ± 35.32 Kpa, respectively. Compared with the 2%HAMA and 10%GelMA SN hydrogels, the compressive elastic modulus of the 10%GelMA/2%HAMA DN hydrogel was significantly improved. The compressive elastic modulus of the GelMA/HAMA/nHap composite hydrogel was even further increased; the compressive elastic modulus of 10%GelMA/2%HAMA/1%nHap hydrogel was more than four times that of the 10%GelMA/2%HAMA DN hydrogel, ten times that of the 10%GelMA SN hydrogel, and twenty times that of the 2%HAMA SN hydrogel.

In Figure 5b, similar to the compressive elastic modulus of the hydrogels, the compressive fracture strength of the GelMA/HAMA DN hydrogels was significantly improved compared with that of the HAMA and GelMA SN hydrogels, the compressive fracture strength of the GelMA/HAMA/nHap composite hydrogel was further increased, and the compressive fracture strength of 10%GelMA/2%HAMA/1%nHap hydrogel nearly reached 1 MPa. The results show that the mechanical strength of the GelMA/HAMA/nHap composite hydrogel was significantly improved by combining the DN strategy and incorporation of nanoparticles, which is nHap in this study.

### 2.4. The Swelling Ratio of GelMA/HAMA/nHap Composite Hydrogels

**Figure 6:**
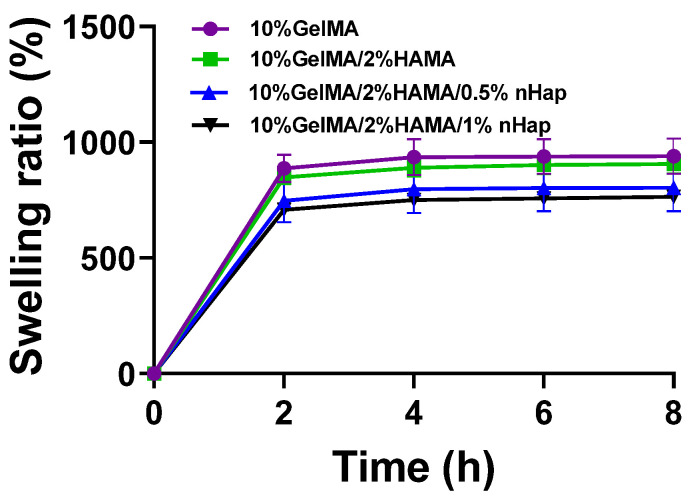
Swelling behavior of the hydrogels in PBS at 37 °C. Data are expressed as mean ± SD (*n* = 3).

Hydrogels with high water content can provide a microenvironment similar to the extracellular matrix and are conducive to the transport of nutrients and expulsion of cellular metabolic wastes, thus promoting cell proliferation and viability [34,35]. According to Figure 6, the hydrogels with different proportions basically reached swelling equilibrium after 6 h. After 8 h, the swelling ratios of the 10%GelMA, 10%GelMA/2%HAMA, 10%GelMA/2%HAMA/0.5%nHap, and 10%GelMA/2%HAMA/1%nHap hydrogels were 939.15 ± 67.96%, 905.19 ± 35.62%, 802.88 ± 91.118%, and 764.39 ± 26.28%, respectively, and their water content were about 90.38%, 90.05%, 88.92%, and 88.43%, respectively. GelMA and HAMA both consist of hydrophilic macromolecules, and the hydrogels composed of a GelMA and/or HAMA crosslinked network can absorb a large amount of water; therefore, the GelMA SN hydrogel and GelMA/HAMA DN hydrogel both possess a high swelling ratio and water content. With the addition of the nHap composite, the swelling ratio and water content of hydrogel decreased due to the non-swelling of nHap but the water content of the composite hydrogels was above 88%. The GelMA/HAMA/nHap composite hydrogel still retained its high water content.

### 2.5. In Vitro Cytocompatibility of GelMA/HAMA/nHap Composite Hydrogels

**Figure 7:**
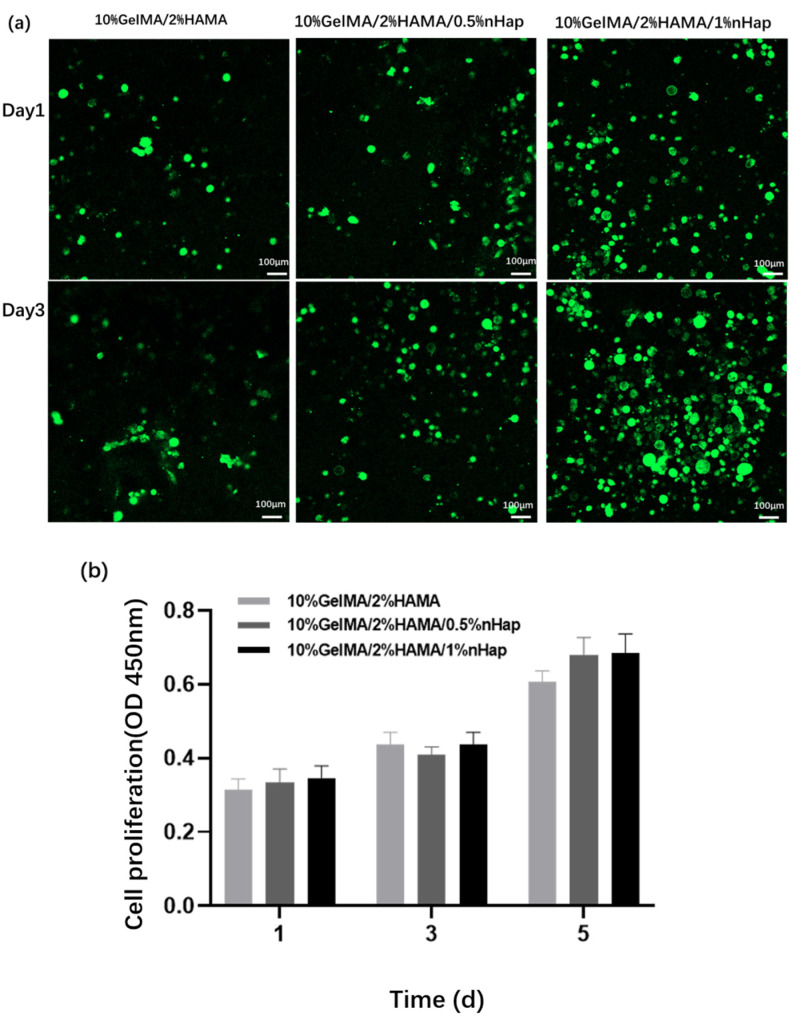
(**a**) Cell viability of BMSCs on the hydrogels, scale bar = 100 μm. (**b**) Cell proliferation of BMSCs on the hydrogels. Data are expressed as mean ± SD (*n* = 4).

The cytocompatibility of the hydrogels was studied by calcein AM staining and CCK-8 determination. BMSCs were inoculated on the surfaces of 10%GelMA/2%HAMA, 10%GelMA/2%HAMA/0.5%nHap, and 10%GelMA/2%HAMA/0.5%nHap hydrogels and continuously cultured for 1 or 3 days. As shown in Figure 7a, after 1 day of culturing, on the surface of the 10%GelMA/2%HAMA, 10%GelMA/2%HAMA/0.5%nHap, and 10%GelMA/2%HAMA/0.5%nHap hydrogels, there were a certain number of BMSCs attached all around, the cell viability was high, and no significant dead BMSCs were observed. After 3 days of culturing, the number of BMSCs on the surfaces of the three hydrogels increased significantly and the number of dead cells was low. After 1 day or 3 days of culturing, the number of BMSCs on the surface of the 10%GelMA/2%HAMA/0.5%nHap hydrogel was obviously higher than that on the surfaces of the other two hydrogels. As shown in Figure 7b for CCK-8 testing, BMSCs on the three hydrogels maintained continuous growth after 5 days of culturing, and the proliferation of BMSCs on the surface of the 10%GelMA/2%HAMA/0.5%nHap hydrogel and the 10%GelMA/2%HAMA/1%nHap hydrogel were both higher than that on the surface of the 10%GelMA/2%HAMA hydrogel. The results of the in vitro cytocompatibility study showed that the GelMA/HAMA/nHap composite hydrogels supported the survival and proliferation of BMSCs on their surfaces, showing good compatibility with BMSC_S_. The GelMA/HAMA/nHap composite hydrogels were fabricated by biocompatible materials using a mild photocrosslinking method. The composite hydrogels had a high swelling ratio and water content due to the hydrophilicity of GelMA and HAMA and became connected porous structures after freeze-drying. These compositions and structural characteristics enable the composite hydrogels to provide a good cellular microenvironment for BMSC_S._

Compared with the GelMA/HAMA DN hydrogel, the proliferation and viability of BMSCs on GelMA/HAMA/nHap composite hydrogels improved significantly. The more nHap content there was, the more BMSCs proliferation followed. The composition, surface properties, stiffness, and mechanical strength of biomaterials affect cell adhesion and proliferation, and stem cell differentiation [36,37,38]. Compared with the GelMA/HAMA DN hydrogel, the compressive elastic modulus of the GelMA/HAMA/nHap hydrogel was enhanced significantly. The proliferation and viability of BMSCs on the surface of the GelMA/HAMA/nHap composite hydrogels were affected by the physical and chemical properties of the hydrogel, and improved due to the increase of mechanical strength and incorporation of nHap. Greater knowledge of the biological mechanism between the hydrogels and BMSCs is needed to investigate further; in vivo animal studies are also needed to evaluate the bone repair ability, biocompatibility, and biodegradability of the composite hydrogels.

## 3. Conclusions

In summary, the GelMA/HAMA/nHap composite hydrogel for bone repair was prepared from GelMA, HAMA, and nHap, which are similar in composition to the bone extracellular matrix, and fabricated by uniformly distributing nHap into the GelMA/HAMA double network followed by photocrosslinking. The results of rheological testing showed that the precursor solution of the GelMA/HAMA/nHap composite hydrogel presented shear thinning, indicating that the precursor solutions of the GelMA/HAMA/nHap composite hydrogel were injectable and may be implanted in vivo by injecting followed by photocrosslinking to form hydrogel..

The mechanical strength of the GelMA/HAMA/nHap composite hydrogel was significantly enhanced by combining the DN strategy and the composite of nHap; the compressive elastic modulus of the composite hydrogel was 20 times that of the HAMA SN hydrogel, and the compressive fracture strength of the composite hydrogel nearly reached 1 MPa. The composite hydrogel retained its high swelling ratio and water content above 88%. The results of the in vitro cytocompatibility study showed that the composite hydrogel supported the survival and proliferation of BMSCs on the surfaces and possessed good compatibility with BMSC_S_. The GelMA/HAMA/nHap composite hydrogel has the potential to be used as an injectable hydrogel for bone repair by minimally invasive implantation.

## 4. Materials and Methods

### 4.1. Materials

Gelatin methacrylate (GelMA, EFL-GM-60), Methylpropenylated hyaluronic acid (HAMA, EFL-HA-40), lithium pheny2,4,6-trimethylbenzoylphosphinate (LAP), and a blue–violet light source (3 W, 405 nm) were obtained from Suzhou Intelligent Manufacturing Research Institute (Suzhou, China). The LIVE/DEAD Cell Viability/Toxicity Kit was obtained from Life Technologies (Carlsbad, CA, USA). Cytotoxicity assay kit (CCK-8), nano-hydroxyapatite (nHap), and Penicillin–Streptomycin Solution were purchased from Solarbio Technology Co. Ltd. (Beijing, China). Australian-sourced fetal bovine serum was purchased from Gibco (Billings, MT, USA).

### 4.2. Preparation of GelMA/HAMA/nHap Composite Hydrogels

#### 4.2.1. Preparation of the Precursor Solutions of the Hydrogels 

The composition of the precursor solution for hydrogels is shown in Table 2. For the 2%HAMA hydrogel, HAMA was dissolved in Phosphate Buffered Saline (PBS) and stirred at 800 rpm, 37 °C for 1 h to prepare the precursor solution. For the 10%GelMA hydrogel, GelMA was dissolved in PBS at 50 °C and stirred at 800 rpm for 1 h to prepare the precursor solution. For the 10%GelMA/2%HAMA hydrogel, HAMA was added to the dissolved GelMA solution followed by dissolving at 37 °C and mixing to prepare the precursor solution by stirring at 800 rpm. For the GelMA/HAMA/nHap hydrogels, a certain amount of nHap was added into a mixture of GelMA and HAMA at 37 °C under stirring at 800 rpm, followed by strong vortexing to obtain a precursor solution with uniformly distributed nHap.

#### 4.2.2. Rheological Testing of the Precursor Solutions of the Hydrogels

The rheological properties of the precursor solution for preparing hydrogels were tested using an MCR 302 modular rheometer (Anton Paar, Graz, Austria) equipped with a plate–plate geometry (10 mm plate diameter). The precursor solution was placed on the test bench and the rotor was lowered to 1 mm. The shear rate was set from “0.01/S” to “100/S”, the temperature was set at 25 °C, and data points were taken every 6 s to detect the relationship between viscosity of the precursor solution and shear rate. To test the relationship between the viscosity of the precursor solution and temperature, a temperature scan under oscillation mode was performed according to linear law. The temperature range was set from 0 to 50 °C with a heating rate of 0.2 °C/s [13].

#### 4.2.3. Preparation of the Hydrogels

The photoinitiator LAP was added into the precursor solutions, prepared as described in Section 4.2.1, by avoiding light and mixed by stirring at 800 rpm, 37 °C. A certain volume of the prepared precursor solutions for the hydrogels was injected into a vessel with certain shape and dimensions at 37 °C away from light and followed by 10 s irradiation with a blue lamp (405 nm) to form the hydrogel.

### 4.3. Characterization of GelMA/HAMA/nHap Composite Hydrogels

The surface chemical elements of freeze-dried GelMA/HAMA hydrogel and GelMA/HAMA/nHap composite hydrogels were analyzed by X-ray photoelectron spectrometer (Thermo Scientific ESCALAB 250Xi, Waltham, MA, USA). The spectrometer used Al Kα microfocusing monochromatic source (ℎν = 1486.6 eV), the obtained electron binding energy was corrected by C1s peak of C 284.8 eV, and the final spectrum was fitted by XPSPEAK41 software. X-ray diffraction (XRD) spectra of nHap and GelMA/HAMA/nHap composite hydrogels were analyzed by X-ray diffractometer (Rigaku SmartLab SE) [32].

The prepared GelMA/HAMA hydrogel and GelMA/HAMA/nHap composite hydrogels were freeze-dried. Samples were then glued to the conductive adhesive and treated with gold spray. The surface and cross-section structure of the freeze-dried hydrogels was observed using a scanning electron microscope (SEM, Zeiss MERLIN compact) at an accelerated voltage of 10 kV. The pore size of the hydrogel was measured from SEM images using ImageJ software [12].

### 4.4. Compressive Strength Testing

The mechanical strength of the hydrogels was studied by conducting a compression test using a universal material testing machine (3345, Instron, Norwood, MA, USA). Each of the 600 μL precursor solutions of 2%HAMA hydrogel, 10%GelMA hydrogel, 10%GelMA/2%HAMA hydrogel, 10%GelMA/2%HAMA/0.5%nHap hydrogel, and 10%GelMA/2%HAMA/0.5%nHap hydrogel were placed into a 48-well plate and photocrosslinked to prepare cylinders with a diameter of 10 mm and thickness of 5 mm. The stress–strain curves of the prepared hydrogel samples were tested at a compression rate of 1 mm/min in the strain region of 0–30%, and the slope of the linear part of the stress–strain curve was calculated to determine the compression modulus of the hydrogels. Additionally, the stress–strain curves were also tested at a compression rate of 1 mm/min until fracture occurred in order to identify the point where cracks began to appear [13].

### 4.5. Evaluation of the Swelling Ratio

For each 100 μL of the precursor solutions of 10%GelMA hydrogel, 10%GelMA/2%HAMA hydrogel, 10%GelMA/2%HAMA/0.5%nHap hydrogel, and 10%GelMA/2%HAMA/0.5%nHap hydrogel, the initial dry weight of each was recorded as W_0_. After formation of hydrogel by photocrosslinking, the hydrogels were soaked in 2 ml PBS (0.01 M, pH7.2–7.4) and swelled at 37 °C for 8 h. The hydrogels were removed from PBS every two hours; then, the water on the surface of hydrogels was carefully picked up with absorbent paper and the hydrogels were measured as W_1_. Swelling equilibrium was reached at the point when the weight of the hydrogels was not changed.

The formula for swelling ratio is as follows [12]:

$$\mathrm{Swelling}\;\mathrm{ratio}=\frac{W_{1}-W_{0}}{W_{0}}\times 100\%$$

### 4.6. In Vitro Cytocompatibility Studies

Rabbit bone marrow mesenchymal stem cells (BMSCs) were used to study the cytocompatibility of the hydrogels. The complete medium containing 89% Dulbecco’s Modified Eagle Medium (DMEM, HyClone), 10% fetal bovine serum (FBS, Gibco), and 1% penicillin/streptomycin (Solarbio) was used as the culture medium for BMSCs [12]. BMSCs were cultured in a humidified incubator at 37 °C with 5% CO_2_, and the culture medium was changed every two days. A sufficient quantity of BMSCs were collected for the following study.

#### 4.6.1. Effects of the Composite Hydrogels on Proliferation Behavior of BMSCs

Each 600 μL precursor solution of 10%GelMA/2%HAMA hydrogel, 10%GelMA/2%HAMA/0.5%nHap hydrogel, and 10%GelMA/2%HAMA/0.5%nHap hydrogel was placed into a 48-well plate followed by photocrosslinking to prepare the hydrogels for in vitro cytocompatibility study. The prepared hydrogels were sterilized using the following method: First, the prepared hydrogel was put into the plate and soaked for 2 h with 75% alcohol of more than 5 times the hydrogel volume. Then, the 75% alcohol-soaked hydrogel was moved to the new plate and the 75% alcohol was replaced by PBS in a volume of more than 5 times the hydrogel volume for 30 min; this process was repeated 4 times. Then, the hydrogel was sealed by PBS and placed in incubator at 37 °C overnight. The BMSCs were digested and adjusted to a cell concentration of 2 × 10^5^/mL by culture medium, and 1 ml BMSCs suspension was dropped on the surface of the hydrogel and cultivated in incubator at 37 °C with 5% CO_2_. After cultivation for 1, 3, and 5 days, the culture medium was removed from the surface of the hydrogel and the hydrogel was cleaned with PBS. The proliferation of BMSCs on the surface of the hydrogel was detected using CCK-8 assay [12].

#### 4.6.2. Effects of the Composite Hydrogels on the Viability of BMSCs

The hydrogel sample was prepared and sterilized following the same method as described in Section 4.6.1. A total 1 ml BMSCs suspension with cell concentration of 2 × 10^5^/mL was dropped onto the surface of the hydrogel and cultivated at 37 °C with 5% CO_2_. After cultivation for 1 and 3 days, the BMSCs on the surface of the hydrogel were stained by Live/Dead Cell Viability/Cytotoxicity Kit (Life Technologies). Images of the stained BMSCs were observed by confocal laser scanning microscope (LSM710, Carl Zeiss, Germany). Living BMSCs showed green fluorescence, whereas dead BMSCs showed red fluorescence [12].

### 4.7. Statistical Analysis

The data were expressed as the mean ± standard deviation (SD). Statistical differences between the groups were tested using one-way analysis of variance (ANOVA) with significance accepted at a *p* value <0.05.

## Figures and Tables

**Table 1:** Surface chemical elements analysis of hydrogels.

Sample	C (%)	O (%)	N (%)	Ca (%)	P (%)
10%GelMA/2%HAMA	64.33	23.55	10.83	0.14	1.16
10%GelMA/2%HAMA/0.5%nHap	68.02	22.42	8.96	0.26	0.35
10%GelMA/2%HAMA/1%nHap	61.11	25.38	11.93	0.67	0.92

**Table 2:** Formulations of the precursor solution for hydrogels.

Hydrogel Formulations	Notations
2%HAMA, 98%PBS	2%HAMA
10%GelMA, 90%PBS	10%GelMA
10%GelMA, 2%HAMA, 88%PBS	10%GelMA/2%HAMA
10%GelMA, 2%HAMA, 0.5%nHap, 87.5%PBS	10%GelMA/2%HAMA/0.5%nHap
10%GelMA, 2%HAMA, 1%nHap, 87%PBS	10%GelMA/2%HAMA/1%nHap

## Data Availability

Data available upon request from authors.
